# Tick-Borne Encephalitis Virus Seropositivity among Tick Infested Individuals in Serbia

**DOI:** 10.3390/pathogens10030301

**Published:** 2021-03-05

**Authors:** Pavle Banović, Dasiel Obregón, Dragana Mijatović, Verica Simin, Srdjan Stankov, Zorana Budakov-Obradović, Nevenka Bujandrić, Jasmina Grujić, Siniša Sević, Vesna Turkulov, Adrian Alberto Díaz-Sánchez, Alejandro Cabezas-Cruz

**Affiliations:** 1 Ambulance for Lyme Borreliosis and Other Tick-Borne Diseases, Pasteur Institute Novi Sad, 21000 Novi Sad, Serbia; Draganav77@gmail.com; 2 Faculty of Medicine, University of Novi Sad, 21000 Novi Sad, Serbia; zorana.budakov-obradovic@mf.uns.ac.rs (Z.B.-O.); nevenka.bujandric@mf.uns.ac.rs (N.B.); jasmina.grujic@mf.uns.ac.rs (J.G.); sinisa.sevic@mf.uns.ac.rs (S.S.); vesna.turkulov@mf.uns.ac.rs (V.T.); 3 School of Environmental Sciences, University of Guelph, Guelph, ON N1G 2W1, Canada; dasieloa@uoguelph.ca; 4 Center for Nuclear Energy in Agriculture, University of São Paulo, Piracicaba, SP 13400-970, Brazil; 5 Department of Microbiology, Pasteur Institute Novi Sad, 21000 Novi Sad, Serbia; Luketic.s@mts.rs (V.S.); stankov.paster@gmail.com (S.S.); 6 Blood Transfusion Institute Vojvodina, 21000 Novi Sad, Serbia; 7 Clinic for Infectious Diseases, Clinical Center of Vojvodina, 21000 Novi Sad, Serbia; 8 Department of Biology, University of Saskatchewan, Saskatoon, SK S7N 5E2, Canada; adiasanz88@gmail.com; 9 Anses, INRAE, Ecole Nationale Vétérinaire d’Alfort, UMR BIPAR, Laboratoire de Santé Animale, F-94700 Maisons-Alfort, France

**Keywords:** TBEV, risk factors, seroprevalence

## Abstract

Tick-borne encephalitis (TBE), caused by the TBE virus (TBEV), is a life-threatening disease with clinical symptoms ranging from non-specific to severe inflammation of the central nervous system. Despite TBE is a notifiable disease in Serbia since 2004, there is no active TBE surveillance program for the serologic or molecular screening of TBEV infection in humans in the country. This prospective cohort study aimed to assess the TBEV exposure among tick-infested individuals in Serbia during the year 2020. A total of 113 individuals exposed to tick bites were recruited for the study and screened for anti-TBEV antibodies using a commercial indirect fluorescent antibody test (IFA) test. Blood samples from 50 healthy donors not exposed to tick bites were included as a control group. Most of the enrolled patients reported infestations with one tick, being *I. ricinus* the most frequent tick found in the participants. The TBEV seroprevalence was higher (13.27%, 15 total 113) in tick-infested individuals than in healthy donors (4%, 2 total 50), although the difference was not significant. Notably, male individuals exposed to tick bites showed five times higher relative risk (RR) of being TBEV-seropositive than healthy donors of the same gender (RR= 5.1, CI = 1.6–19; *p* = 0.007). None of the seropositive individuals developed clinical manifestations of TBE, but the first clinical-stage of Lyme borreliosis (i.e., erythema migrans) was detected in seven of them. Potential TBEV foci were identified in rural areas, mostly in proximity or within the Fruška Gora mountain. We conclude that the Serbian population is at high risk of TBEV exposure. Further epidemiological studies should focus on potential TBEV foci identified in this study. The implementation of active surveillance for TBEV might contribute to evaluating the potential negative impact of TBE in Serbia.

## 1. Introduction

The tick-borne encephalitis (TBE) is a zoonotic infectious disease caused by the TBE virus (TBEV) which is a member of the genus *Flavivirus*, family Flaviviridae [1]. The disease is most frequently reported in the central and eastern regions of Europe and Asia, where *Ixodes* ticks are the main vectors [2]. Based on phylogenetic analysis of the E protein, the TBEV has been classified into three subtypes, namely European (TBEV-Eu), Far-Eastern (TBEV-FE), and Siberian (TBEV-Sib) [3]. The circulation of TBEV in Europe is restricted to natural foci, where the most important vector is *Ixodes ricinus* and the main vertebrate reservoirs are small rodents of the genera *Apodemus* and *Myodes* [4,5]. The establishment of TBEV foci requires many factors to be fulfilled, such as terrain and climate conditions suitable to maintain an adequate population of reservoirs host and vectors, as well as the presence of large animals which provide blood meals for questing infected ticks [6,7]. TBEV foci are dynamic due to changes in the foci-promoting factors over time [8].

The clinical manifestations of TBE include non-specific symptoms (e.g., febrile illness) to severe inflammation of the central nervous system (CNS). The higher risk of TBEV infection occurs in individuals exposed to tick bites within TBEV foci [6,9]. Excretion of viral particles from the tick to the host begins immediately after tick attachment and lasts until tick detachment [9]. Human infection can also occur after ingestion of food contaminated with TBEV. Oral infection occurs frequently after ingestion of unpasteurized milk or dairy products obtained from TBEV-infected cows, sheep, or goats [10]. The oral route of infection is considered to be of lower epidemiological importance as it occurs rarely and is highly localized [11]. According to European Centre for Disease Prevention and Control (ECDC), 3212 cases of TBE were reported in EU/EEA countries in 2018, with a notification rate of 0.6 cases per 100,000 citizens [12].

The endemic areas of TBE in Europe are expanding outside of the territories (i.e., Central European countries, Baltic countries, and Russia) traditionally considered at high-risk for TBEV infection. The incriminated reasons include climate change, population movement, and growth, modifications of landscapes and natural habitats, as well as improved surveillance and diagnostic techniques [13]. One of the most striking examples of TBEV expansion is the recently reported presence of the TBEV-Sib subtype in the Western Balkans, a region where only the TBEV–Eu subtype had been reported [14]. Serbia is located at the crossroads of Central and Southeast Europe and it is in proximity to the countries with TBE endemic regions (i.e., Croatia, Hungary, and Slovenia) [4,10,15]. The Serbian landscape is diverse, with plain northern parts (i.e., Vojvodina province) covering the southern edges of the Pannonian Plain, and the rest of the country covers the central parts of the Balkan Peninsula, mainly consisting of hills and mountains, interspersed with numerous rivers and creeks. TBEV was first detected in Serbia in 1972 when one TBEV-Eu isolate was detected in a hard tick collected in the Pešter plateau [16]. More than 45 years later, two additional TBEV-Eu strains were isolated from *I. ricinus* collected at Fruška Gora mountain and rural suburbs of Belgrade [17].

Despite TBE is a notifiable disease in Serbia since 2004, there is low awareness about this disease among the medical practitioners of the country. And there is no active TBE surveillance program oriented toward serologic or molecular screening of patients with signs and symptoms related to viral infection of CNS [18,19]. New cases of TBE in Serbia are periodically detected in only one medical facility, whereas diagnosis is performed only through serological method (i.e., via ELISA assays) [18]. To evaluate the potential TBEV exposure risk in humans in Serbia, we conducted a prospective cohort study during the year 2020 to assess the countrywide seroprevalence of TBEV as well as to identify the risk groups and foci associated with the presence of TBE in Serbia.

## 2. Materials and Methods

### 2.1. Study Design

Individuals referred from local Community Health Centers and Medical Emergency Centers presented to the Pasteur Institute Novi Sad with cases of tick infestation. Follow-up visits to the institute were organized for at least 2 months after tick removal. Patients were invited to participate in the study, regardless of clinical findings related to tick infestation. After receiving the informed consent of participation from each patient, or patient’s caretakers (in case of underage individuals), a blood sample was collected and the serum analyzed for reactivity against TBEV antigens. The clinical history of each patient archived at the institute was searched for anamnesis data related to specific or non-specific manifestations of TBE. Only individuals that reported no previous vaccination against TBEV were recruited. Demographic data such as gender, age, and settlement (urban/rural) were registered for each patient (Table S1).

### 2.2. Selection of Healthy Blood Donor Samples

As a control group, we used sera samples from healthy blood donors with a regional distribution overlapping that of the majority of enrolled patients. South Bačka District is located in proximity to the north side of Fruška Gora mountain and the Danube River. After receiving the informed consent, each blood donor filled in a questionnaire with data related to his/her residency, age, gender, profession/occupation, hobbies, immunizations against TBE, CNS inflammatory diseases, and previous tick bites (Table S2). Their blood samples were independently analyzed in Blood Transfusion Institute of Vojvodina for the presence of Human Immunodeficiency Virus (HIV) antigens, HIV genetic material, anti-HIV antibodies, hepatitis C virus (HCV) antigens, HCV genetic material, anti-HCV antibodies, and anti-*Treponema pallidum* antibodies as part of routine screening of blood donors. Only the sera samples from donors with negative results on screening tests were included in the examination of anti-TBEV antibodies.

### 2.3. Tick Collection and Classification

All ticks collected from patients were identified regarding species, sex, and life stage, based on morphological features and standard taxonomic keys described by Estrada-Peña et al. [20]. Patients provided information about the suspected location where the ticks were acquired. When the patient was unsure about the time elapsed between tick bite and detection, the scutal and coxal indexes were calculated and used to assess the tick-feeding period [21]. Patients were encouraged to report immediately new episodes of tick infestation on them, regardless of the date scheduled for the next medical examination at Pasteur Institute Novi Sad.

### 2.4. Blood Sample Collection and Detection of Anti-TBEV Antibodies

For each patient, 3 mL of blood were extracted via venipuncture in BD Vacutainer^®^ SST™ Tubes (BD, Franklin Lakes, NJ, USA) at least 8 weeks after the removal of the tick. Blood was allowed to clot inside the vial and sera samples were extracted after centrifugation at 2000× *g* for 10 min. Sera samples were inactivated at 56 °C. Extracted sera were then used anti-TBEV IgG detection using TBEV particles in a commercial immunofluorescence assay (IFA) kit (Euroimmun, Lübeck, Germany; Cat. No. FI 2661-1005). The assay was carried out following the manufacturer’s instructions. Positive fluorescence reactions at 1:10 sera dilution were considered positive. Serology results were interpreted in a qualitative form (i.e., positive or negative). Therefore, in the case of seropositive finding no further sera dilution was performed. Fluorescence was analyzed on a microscope Leica DM 3000 (Leica, Wetzlar, Germany) with a light source from a mercury bulb using an N2.1 filter (Leica, Wetzlar, Germany) with an excitation wavelength of 515–560 nm.

### 2.5. Data and Statistical Analysis

To identify risk groups for exposure to TBEV, we analyzed patient clinical findings, and demographic data such as gender, age, and settlement (urban/rural). Age groups were formed as followed: children (1–10 years of age), teenagers (11–20), adults (21–64), and seniors (≥65). The association between disease and risk factors was tested using the relative risk (RR) measurement [22]. Chi-square (χ^2^) association test, with Yates’s correction, was performed to avoid overestimation of statistical significance because of small numbers of cases. Fisher exact probability test was performed when any of the groups under comparison had less than five cases to be included in a contingency table. The analyses were performed on the statistical webtool VassarStats (http://www.vassarstats.net) (accessed on 20 January 2021). To reveal territory with possible TBEV foci, we integrated serologic results of each patient with geographic location wherein tick was acquired. To access probable tick acquiring location, the patients were asked to recall localities where he/she performed any activities with risk for an encounter with ticks in the time range given by extrapolation of scutal and coxal indexes [21]. For demographic data analysis, we used χ^2^ test using software SPSS Statistics 25 (IBM, Armonk, NY, USA). Statistically significant differences were considered when *p* < 0.05.

## 3. Results

### 3.1. Patient Enrollment

A total of 676 patients who reported to the Pasteur Institute Novi Sad during the year 2020 were evaluated for their inclusion in the study. At the moment of the visit to the institute, 618 patients (91.42%, total 676) had ticks still attached to, or recently removed from, the skin. Tick infestation could not be confirmed in 58 (8.58%, total 676) patients and therefore they were not considered for the study. After 2 months of follow-up, 187 (27.66%, total 676) patients were asked to enroll in the study, and 113 of them (16.71%, total 676) agreed to participate. None of the enrolled patients reported previous infections or symptoms associated with the central nervous system (CNS) or previous immunizations against TBEV or Yellow fever. The distribution of patients and healthy donors enrolled in the study is shown in Table 1.

### 3.2. Tick Infestation among Enrolled Patients

Of the 113 patients enrolled in the study, 105 (92.92%), 7 (6.19%), and 1 (0.88%) reported infestations with one, two, or three ticks, respectively. Among the 121 ticks collected from the patients, we identified *Ixodes ricinus*, *Rhipicephalus sanguineus*, and *Dermacentor reticulatus* in 117, 2, and 2 patients, respectively. All collected *R. sanguineus* and *D. reticulatus* were adult female ticks, while collected *I. ricinus* included 10 larvae, 55 nymphs, 51 adult females, and 1 adult male (Table S1). Significant differences were found in the frequency of *I. ricinus* stages found in the patients (nymphs and adults vs. larvae χ^2^(1) = 34.231, *p* < 0.001; larvae vs. nymphae χ^2^(1) = 31.154, *p* < 0.001 and larvae vs adults χ^2^(1) = 32.061, *p* < 0.001). We found no significant difference in the frequency of infestation with adults compared to nymphal tick stages (χ^2^(1) = 0.009, *p* = 0.924). Only one blood donor (2%, 1 total 50) reported tick infestation in the six months previous to the study, whereas the majority (62%, 31 total 50) reported having hobbies that include activities in nature (Table S2).

### 3.3. Clinical Course in Patients Infested with Ticks

None of the 113 patients developed clinical manifestations of TBE. Seven patients (6.19%, 7 total 113), however, developed the first stage of Lyme borreliosis (i.e., erythema migrans). These 7 patients reached complete recovery after a 2-week treatment with amoxicillin or doxycycline. One 16-year-old boy developed a mild fever of 37.7 °C followed by fatigue, loss of appetite, and chills, two days after the removal of the tick. The signs and symptoms lasted four days in this patient, followed by a complete recovery. Eight weeks later, the patient was tested for anti-TBEV IgG antibodies and the serum sample was seropositive. The tick removed from the 16-year-old boy was an *I. ricinus* nymph, with an estimated feeding period of four days. The Rtanj mountain (Boljevac municipality) was reported as the suspected location where the tick bite occurred.

### 3.4. TBEV Seroprevalence in Patients and Healthy Donors

TBEV Seroreactivity was higher (13.27%, 15 total 113, Table S1) in tick-infested individuals than in healthy donors (4%, 2 total 50, Table S2), although no significant difference in the seroprevalence values of these two groups was found (χ^2^(1) = 2.276, *p* = 0.131). Notably, we found significant difference (χ^2^(1) = 11, *p* = 0.001) in TBEV seroprevalence when male (41.07%, 23 total 56) and female (12.07%, 7 total 57) patients were compared, with a significant higher relative risk (RR) of TBEV exposure for male individuals (RR = 3.3, CI = 1.6–7.2; *p* = 0.001). Moreover, no significant difference was found in TBEV seroprevalence (χ^2^(1) = 0.57, *p* = 0.45) or RR (RR = 2.8, CI = 0.56–17; *p* = 0.45) between groups of patients living in urban (14.8%, 14 total 94) or rural (5.26%, 1 total 19) areas. Concerning the age groups, the highest seroprevalence was detected in children (17.9%, 5 total 28) and seniors (21.4%, 3 total 14), compared to teenagers (7.69%, 1 total 13) and adults (10.3%, 6 total 58). Despite higher seropositivity values among children and seniors, no significant difference was associated with the age groups included in this study (χ^2^(3) = 2.1036, *p* = 0.551).

**Figure 1:**
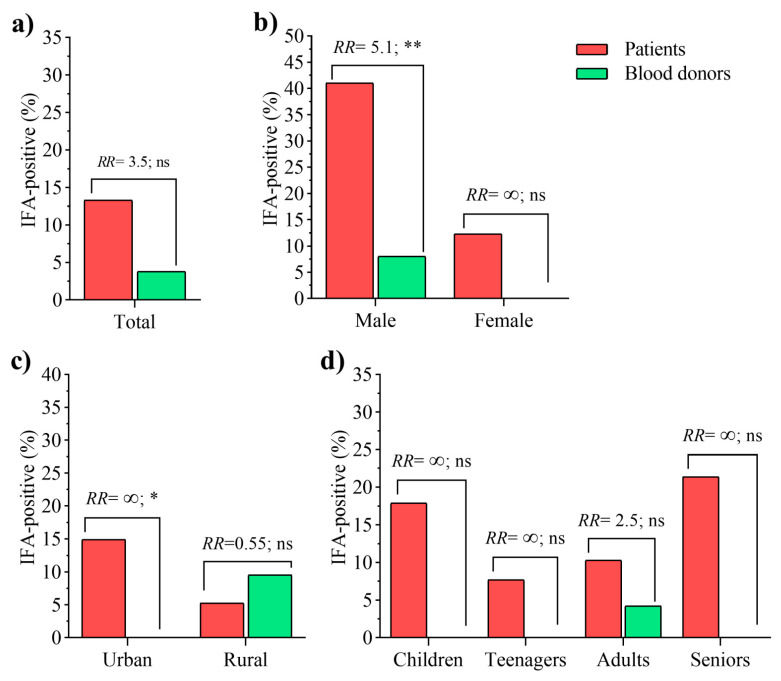
Seroprevalence of tick-borne encephalitis virus (TBEV) in patients and healthy donors according to gender, type settlement, and age. (**a**) Comparison of total prevalence values between the studied groups. (**b**) Comparison between the groups of males, and also between females from patients and healthy donors. (**c**) The patients and donors were also compared when inhabiting in urban, or from rural areas. (**d**). Comparisons were performed in each age group as well, despite the no children or seniors individuals were present in the cohort of healthy donors. RR: relative risk, indicate the probability of an individual in the groups of “Patients” to be seropositive to TBEV. RR = ∞ (undefined) when the prevalence value equals 0 in a group. The significance of the association was also tested by Fisher exact test (* *p* < 0.05; ** *p* < 0.01; ns, non-significant).

When compared with blood donors, the RR analysis revealed that individuals infested by ticks in Serbia are three times more likely to be TBEV-seropositive than healthy donors (Figure 1a), however, the comparison rendered no statistical significance (RR = 3.5, CI = 0.91–13; *p* = 0.10). Furthermore, patients and healthy blood donors were compared considering the risk factors, gender (male vs. female, Figure 1b) and settlement (rural vs. urban, Figure 1c) and age groups (children, teenagers, adults, and seniors, Figure 1d). Of note, male individuals exposed to tick bites have a five times higher risk of being TBEV-seropositive than healthy donors of the same gender (RR = 5.1, CI = 1.6–19; *p* = 0. 007) (Figure 1b). It is noteworthy that no seropositive case was found in the healthy female group (Figure 1b), suggesting that females exposed to ticks may also be prone to being TBEV-seropositive, despite the lack of statistical significance in this comparison (12% vs. 0%; RR= ∞, CI = 0.88–∞; *p* = 0. 16). The settlement type was revealed as an important risk factor of TBEV exposure. Particularly, seropositive cases were only found among patients of urban areas, and the difference in patients and donors TBEV seroprevalence was significant (RR= ∞, CI = 1.2–∞; *p* = 0. 04). However, no difference was observed between blood donor (9.52%, 2 total 21) and patients (5.26%, 1 total 19) from rural areas (RR = 0.55; CI = 0.07–3.9; *p* = 0.93, Figure 1c). No age category was associated with the risk of being seropositive to TBEV (Figure 1d). In adults, despite the seroprevalence was higher in patients (10.3%, 6 total 58) than in healthy donors (4.1%, 2 total 48), no significant RR was found between the groups (RR = 2.5; CI = 0.61–10; *p* = 0.41).

### 3.5. Identification of Potential TBEV Foci

**Figure 2:**
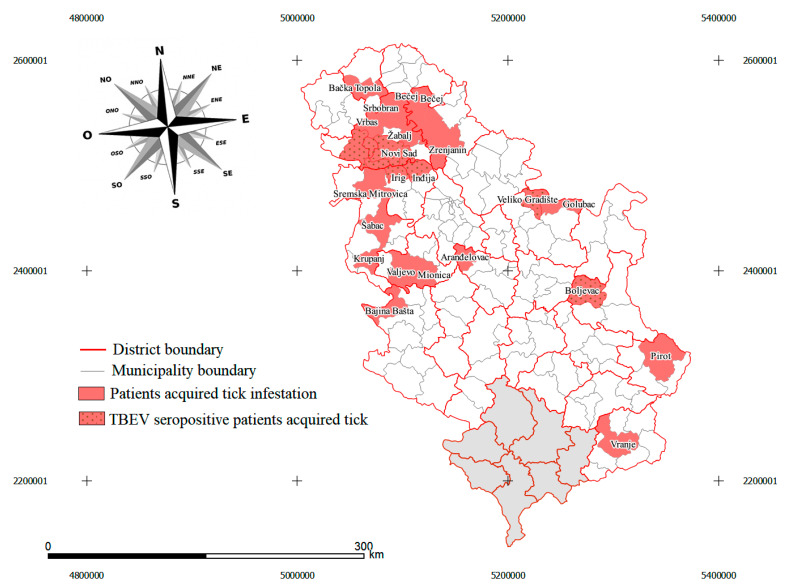
Map of Serbia with administrative division at Nomenclature of Territorial Units for Statistics (NUTS) 2 level. Municipalities, where patients acquired tick infestation, are colored with red. Municipalities wherein TBEV seropositive patients acquired tick infestation are marked with black dots. In northern Serbia, a pattern can be observed as almost all municipalities where the Fruška Gora mountain spreads are marked with red stripes (Inđija, Irig, Novi Sad, Beočin, Sremski Karlovci, Bačka Palanka). In central Serbia, Rtanj mountain (Boljevac municipality) and the territory near the Danube River in the Veliko Gradište municipality with the possible presence of TBEV foci. The map was generated by using QGIS v3.12 (QGIS Development Team, 2020).

The majority of seropositive patients reported that they were exposed to tick bites in rural areas (66%, 10 total 15), mostly in the proximity of, or within, the Fruška Gora mountain including the localities of Bukovac, Čortanovci, Popovica, Rakovac, Tekije, rural part of Sremska Kamenica and Obrovac (Figure 2, Table S1). Two other seropositive patients reported Rtanj mountain and Veliko Gradište as the location where they were bitten by ticks. From seropositive patients with tick bites in urban areas (34%, 5 total 15), four were exposed to ticks in Novi Sad, and one was exposed to ticks in the urban part of Sremska Kamenica town. We found no statistically significant differences regarding localities where tick exposure was reported (χ^2^(2) = 1.667, *p* = 0.197). From the total sample of healthy blood donors acquired in 6 municipalities, we only found seroreactivity in 2 persons (4%, 2 total 50) and both resided in Obrovac village within Bačka Palanka municipality (Table S2).

## 4. Discussion

In the last three decades, the number of TBE cases has increased in Central and Eastern Europe, where this disease is the most relevant tick-borne CNS-infection [2]. On the other hand, the geographic limits of TBEV endemic regions are expanding in Europe [2]. More than 3000 TBE cases are reported annually in 27 European countries considered as endemic for TBEV [23]. The possible reason for the increased incidence and establishment of new TBEV foci is the rising temperatures due to climate change. Higher temperatures favor the increase in vector populations and animal reservoirs, migration of vectors to higher altitudes, and extended time that people spend in outdoor activities in parks and gardens [24,25,26]. In this paper, we have presented original data on the first TBEV seroprevalence study for patients infested with ticks in Serbia. The serum neutralization test (SNT) is the gold standard assay for TBEV diagnosis [27], but this assay is not currently performed in Serbia. Instead, the use of enzyme-linked immunosorbent assay (ELISA) specific for the detection of anti-TBEV IgM and IgG is regularly described for serology testing and etiological confirmation [18]. The main drawback of using IFA or ELISA assays for TBEV seroprevalence examination is the occurrence of cross-reactivity with antibodies against related flaviviruses such as the West Nile Virus (WNV) and Usutu Virus (USUV), which have been reported in Serbia [28]. To overcome the limitation of testing TBEV seroprevalence by IFA and reduce the possible bias associated with it, in the present study we introduced a control group that consisted of sera samples from blood donors living in the same municipalities as patients infested with ticks (i.e., municipalities at the northern side of the Fruška Gora mountains). Therefore, if seroreactivity occurs as a consequence of cross-reaction with antibodies against other flaviviruses, it is expected to be equally present in blood donors as in patients infested with ticks. One limitation of the present study is the low number of samples within each risk group category included in the relative risk analysis (Figure 1). This limitation might explain the lack of statistical significance in some of the analyses. For example, the low number of patients recruited in the category “teenagers” (i.e., 2), and the lack of children and seniors among the healthy donor samples, did not allow for a robust assessment of the distribution of TBEV seroprevalence in these age categories. Another limitation is the exclusive use of anti-TBEV IgG seroreactivity test, which may have failed to detect early TBEV infections. For example, in the case of a 16-year-old boy presenting with a fever after the tick bite, we were not able to conclude TBEV infection as IgM antibodies were not examined promptly and the tick was not tested for the presence of TBEV RNA. Future studies should include anti-TBEV IgM and IgG seroreactivity tests in patient samples and TBEV RNA detection in paired tick samples.

In the present study, TBEV seroprevalence rates found in patients exposed to tick bites (13.27%) and healthy blood donors (4%) were lower than those reported from some endemic regions of Europe. For instance, TBEV seroprevalence in non-vaccinated individuals from endemic regions in Poland was as high as 81% [29], while anti-TBEV antibody prevalence in forestry workers from southwest Germany was up to 27% in areas with no reports of clinical cases [30]. Previous serological investigations performed during the years 2014 and 2015 in healthy individuals in northern Serbia confirmed the presence of anti-TBEV antibodies using ELISA assays [28]. The first study reported the presence of IgG antibodies against TBEV in 7.9% (8/101) of healthy individuals in the region of South Bačka, whereas there were no seropositive findings (0/80) in the Nišava District [28]. The TBEV seroprevalence rate herein reported in blood donors differs from the results published in 2014 (4% vs. 7.9%), which could be due to the small sample size in both studies, as well as differences in the inclusion criteria of the study target population. The second study examined the presence of anti-TBEV antibodies in 267 human sera samples from patients hospitalized in the Clinic for Infectious Diseases within the Clinical Center of Vojvodina [17]. Anti-TBEV IgG were detected in six patient samples (2.24%), where five of them were eliminated due to cross-reactivity with WNV and USUV antigens, resulting in an overall seroprevalence of 0.37% [17]. Unfortunately, Potkonjak et al. [17] reported no data about residency, lifestyle as risk factors, earlier tick exposures, or the precise reason why enrolled patients were hospitalized. For that reason, reported seroprevalence cannot be easily compared with a healthy population (i.e., blood donors), neither with a population previously infested with ticks. Circulation of TBEV within the local population was also reported in the City of Belgrade, where a group of authors published case series of 10 TBE patients during the period between June and August of the year 2017 [17]. Detection of TBE cases in Belgrade is in accordance with previously reported results where TBEV was found in ticks from the same area. Together, these studies highlight the importance of field tick research for public health and overall preparedness for neglected tick-borne diseases such as TBE in Serbia.

Despite no age category was associated with the risk of being seropositive to TBEV, we observed that the majority of TBEV seropositive individuals in TBEV foci in Serbia were seniors and children. The explanation for the lower seroreactivity in adult and teenager age groups may be that TBEV was not widely present in this territory earlier, as it is known that the number of TBEV foci in one territory can fluctuate over the years [31]. According to our results, teenagers and adults have a relatively lower chance of being exposed to TBEV, while children and seniors (i.e., persons who are currently spending the most time in parks and nature) have higher chances for TBEV exposure. This finding is in accordance with a previous study that identified children and seniors as the age group with a high risk of tick bites, most probably since they have the most free time at their disposal [32]. Thus, our results do not concur with those by Toczylowski et al. that reported a lower TBEV seropositivity in children compared to teenagers in Poland [33]. The differences in TBEV seroprevalence in children between the studies could be due to dissimilarities in the study design and cohort selection. Specifically, all the patients, including children, included in our study were previously exposed to tick bites; while in the study by Toczylowski et al. [33] children with CNS infection of different causes including enteroviruses, varicella zoster virus, and tick-borne infections were included.

Due to the high occurrence of asymptomatic course of TBEV infection, it was not unexpected that none of the 17 seropositive patients of our study showed, or developed, signs/symptoms of TBEV infection. Furthermore, by using TBEV-specific IgG we were able to detect only past TBEV infections and therefore any symptoms/signs of infection might have gone unnoticed or misdiagnosed. This finding is in accordance with previous studies reporting that in more than 70% of TBEV-positive cases the infection is asymptomatic [19]. Moreover, the only TBEV variant detected up to date in Serbia is the TBEV-Eu subtype, which has been described as less virulent compared to subtypes TBEV-Sib and TBEV-Fe [17,34].

*I. ricinus* was the predominant tick species found in the study, accounting for 117 out of 121 (96.69%) specimens. This finding was expected since *I. ricinus* is the main tick vector for the TBE virus in Western Europe [35], and the most widespread tick species in Serbia [36,37,38]. All active developmental stages of *I. ricinus*, including larvae, nymphs, and adults, can be infected with TBEV and transmit the virus, throughout the whole life cycle to animal reservoirs and humans [39]. For this reason, based on the obtained results and previous studies, we hypothesized that in the sampled region the TBEV is transmitted by tick bites. Moreover, in patients exposed to tick bites, a significantly higher frequency of *I. ricinus* infestation was found in the nymph and adult tick species stage than the larval stage. This finding is explained by the tick questing activity which during the larvae developmental stage prefers to inhabit in leaf litter and the lower vegetation layers where predominantly parasitizes small mammals such as the wood mice *Apodemus sylvaticus* and bank voles *Myodes glareolus* [40]. There have been reported behavioral changes in TBEV infected ticks due to virus penetration in the tick nervous system which makes virus-infected ticks, mainly nymphs and adults may attack hosts more often and aggressively than non-infected individuals [41]. Through the mapping of tick acquiring locations in patients that had positive TBEV serology findings, we were able to identify new possible high-risk locations for exposure to the virus in Serbia. As shown above, most of those locations are in proximity to Fruška Gora mountain and include the territory of six municipalities. This finding is in accordance with the research of Potkonjak et al. (2017) where the TBEV-Eu subtype was identified in ticks in the Fruška Gora mountain [17]. Although none of the patients reported tick bites within the locality where Potkonjak et al. (2017) previously isolated TBEV, it is possible that there are more TBEV clusters scattered through the whole mountain range. Using the same mapping technique of tick-acquiring locations, for the first time, we hypothesize the existence of TBEV natural foci in the Rtanj mountain (Boljevac municipality) and the territory near the Danube River in the Veliko Gradište municipality (near the Romanian border). Although TBEV infection belongs to the list of reportable diseases in Serbia there are no reported cases because the diagnostics are not performed routinely. The public and medical awareness on the epidemiology of TBE is changing through Europe since TBEV infection appears to be an increasing local as well as a global public health problem with a significant economic impact on society. Moreover, we proposed several localities to be examined for the presence of TBEV natural foci. Considering the epidemiological context of Serbia, we conclude that TBEV could be a neglected cause of CNS diseases in the country, and therefore a TBEV surveillance system is needed throughout the country.

## 5. Conclusions

Results presented here indicate that exposure to tick infestation in the Serbian population is associated with a high risk of TBEV infection. Male individuals exposed to tick infestation are at a significantly higher risk of TBEV infection in Serbia. Notably, the study revealed that human settlements in urban areas are not exempt from TBEV exposure risk. Future studies on this topic will benefit from including a higher number of samples in the different categories analyzed in this study. Due to possible cross-reactivity of anti-TBEV IgG with WNV and USUV antigens [17] and the presence of WNV and USUV [28] in Serbia, we recommend the use of additional TBEV-specific antibody detection systems such as the neutralization assay in future studies. In addition, the implementation of an active surveillance system for TBEV in patients with signs of viral CNS infection will improve TBE incidence assessment in Serbia.

## Figures and Tables

**Table 1:** Distribution of the analyzed samples according to risk groups.

Risk Group	Patients	Blood Donors
Gender		
Male	56	25
Female	57	25
Total	113	50
Age		
Children	28	0
Teenagers	13	2
Adults	58	48
Seniors	14	0
Total	113	50
Settlement		
Urban	94	29
Rural	19	21
Total	113	50

## Data Availability

No new data were created or analyzed in this study. Data sharing is not applicable to this article.

## Supplementary Materials

The following are available online at https://www.mdpi.com/2076-0817/10/3/301/s1, Table S1: Demographic data for patients with tick infestation, Table S2: Demographic data for healthy blood donors.
